# Neuralgia vs. Tooth-Ache

**Published:** 1850-04

**Authors:** S. M. Shepherd

**Affiliations:** Petersburg, Va.


### ARTICLE IV.

### *Neuralgia* VS. *Tooth-ache.* By S. M. SHEPHERD, D. D. S. *of Petersburg, Va.*

Neuralgia has become a very fashionable disease now-a-days, and many persons suffer long and severely, and ransack the whole materia medica in search of remedies; and finally an examination of the teeth is thought of, the very first thing that should have been done. In nine cases out of ten of supposed neuralgia, the extraction of some badly decayed tooth, which the suffering individual knows ought to have been out more than a year ago, perhaps, would cause a subsidence of all symptoms of neuralgia.

As a prominent example of the above, I am induced to report the following case: Miss C. W., a resident of this town, of delicate constitution, was attacked with severe pain in the right side of the head, neck and shoulder, about twelve months ago; and from the severity of the pain, and other circumstances attending it, she came to the conclusion that it was neuralgia; and by concurrence with her medical adviser, her opinion was confirmed. She used, therefore, all possible remedies for that disease without success. In the meantime her attacks were growing more frequent and more severe; and for the last two or three months, they occurred daily at precisely five o'clock in the afternoon, and continued with the most intense severity until midnight; when the pain would begin gradually to subside, growing less and less until she was perfectly easy. These daily attacks came on with such perfect regularity, to use her own words, "five o'clock was a terror to her before it came." At this stage of the disease she was in the city of Baltimore, whether in search of medical advice or not, I do not know; but while there, she consulted Dr. B., an eminent physician of that city; and he advised her to have her teeth examined, intimating that they might be involved; he gave her at the same time, a prescription for neuralgia, to be used in case the teeth were not at fault. With this advice she returned home, and sent for me, and related to me substantially, what I have stated above. I examined her teeth and found the inferior wisdom tooth of the right side decayed to the nerve, and I gave it as my opinion that all her "neuralgia" originated there; I therefore advised its immediate extraction to which she assented. The first day after the tooth was extracted she had very little pain, the next still less, and the third none at all.

Thus a perfect cure was affected, of what perhaps nineteen out of twenty of our very best physicians would have pronounced neuralgia, without once thinking of the teeth, by the simple extraction of a bad tooth.

I do not offer the above as a case of rare occurrence; I have often met with such in the course of my dental practice, as doubtless dentists in general have; and I cannot account for the fact, that physicians so generally prescribe for neuralgia, without once thinking of the teeth, when there is so striking a similarity to true neuralgia in many cases of tooth-ache. In the case above, there were some striking peculiarities, which would have been, perhaps, sufficient to screen the most vigilant from the charge of superficiality in the examination of his patients, though he might have forgotten the teeth. The duration, the regular increase of pain, the extent to which the system was affected, and when the attacks became daily, the perfect uniformity as to the time of commencement, together with the nervous temperament of the subject, were all circumstances well calculated to mislead the judgment; and yet this proved to be a case of tooth-ache, a fact which might have been proved just as easily in its very commencement, if an examination of the teeth had been once thought of as a matter of any consequence.

